# Identification and Genome Analysis of *Vibrio coralliilyticus* Causing Mortality of Pacific Oyster (*Crassostrea gigas*) Larvae

**DOI:** 10.3390/pathogens9030206

**Published:** 2020-03-11

**Authors:** Hyoun Joong Kim, Jin Woo Jun, Sib Sankar Giri, Cheng Chi, Saekil Yun, Sang Guen Kim, Sang Wha Kim, Se Jin Han, Jun Kwon, Woo Taek Oh, Sung Bin Lee, Ji Hyung Kim, Se Chang Park

**Affiliations:** 1 Laboratory of Aquatic Biomedicine, College of Veterinary Medicine and Research Institute for Veterinary Science, Seoul National University, Seoul 08826, Korea; hjoong1@nate.com (H.J.K.); giribiotek@gmail.com (S.S.G.); arseidon@naver.com (S.Y.); imagine5180@gmail.com (S.G.K.); kasey.kim90@gmail.com (S.W.K.); sejin.n@daum.net (S.J.H.); kjun1002@snu.ac.kr (J.K.); mike0202@snu.ac.kr (W.T.O.); lsbin1129@naver.com (S.B.L.); 2 Department of Aquaculture, Korea National College of Agriculture and Fisheries, Jeonju 54874, Korea; advancewoo@hanmail.net; 3 Laboratory of Aquatic Nutrition and Ecology, College of Animal Science and Technology, Nanjing Agricultural University, Nanjing 210095, China; chicheng0421@126.com; 4 Infectious Disease Research Center, Korea Research Institute of Bioscience and Biotechnology, Daejeon 34141, Korea

**Keywords:** *Vibrio* species, Pacific oyster (*Crassostrea gigas*) larvae, genome analyses, ChrI, ChrII, SNUTY-1

## Abstract

*Vibrio coralliilyticus* is known as a coral pathogen that also infects marine bivalve larvae worldwide. It is considered to be one of the major constraints in artificial marine bivalve seed production as it causes mortality. In this study, we first isolated and characterized a high virulent of *V. coralliilyticus* designated as SNUTY-1 that was the cause of Pacific oyster larvae mortality in Korea. In the pathogenicity test, exposure to 2.14 × 10^5^ CFU/mL for 24 h caused mortality to 88.65 ± 2.4% of the tested healthy Pacific oyster larvae. SNUTY-1 showed anti-microbial resistance to β-lactams, such as penicillins, cephalosporins, and carbapenems. We sequenced and assembled the complete genome of SNUTY-1 (5,842,676 bp), consisting of two chromosomes (Chr I and Chr II) and two plasmids (pSNUTY1 and pSNUTY2). The COG functional analysis confirmed that Chr I had more genes associated with basic cellular functions in comparison to Chr II. The results of the phylogenetic trees based on OrthoANI values indicated that the SNUTY-1 was closely related to *V. coralliilyticus* strains. SNUTY-1 had a unique plasmid (pSNUTY2), which could mean that the Korean isolate is different from other sequenced *V. coralliilyticus* strains from different geographical origins. Toxic proteins such as cytolysin/hemolysin and extracellular metalloprotease genes were encoded on Chr I and Chr II of SNUTY-1. These data facilitate the control of *V. coralliilyticus* infections in aquaculture by providing valuable insights into the biodiversity of this organism and valuable information for the study of virulence factors.

## 1. Introduction

Oysters are the most consumed shellfish worldwide and account for the largest commodity in the bivalve aquaculture industry. The Republic of Korea is a major producer of Pacific oyster (*Crassostrea gigas*). In 2017, it produced 315,255 tons out of the total 639,030 tons (49.33%) of Pacific oysters produced worldwide [1]. However, since the middle of the 2000s, there have been perpetual occurrences of mass mortality owing to bacillary necrosis in Korean oyster seedling production hatcheries. *Vibrio* sp. has been speculated to be the cause, however, there have been no definite investigation until now. 

The genus *Vibrio* is composed of ubiquitous aquatic bacteria, including diverse members of planktonic and animal-associated microbial communities [2]. Several organisms in this genus, such as *V. alginolyticus*, *V. anguillarum*, *V. splendidus* biovarⅡ, *V. tubiashii*, and *V. coralliilyticus,* have been associated with mass mortalities in nursery cultures of juvenile bivalves at oyster hatcheries worldwide [3,4,5,6,7]. Although all of the abovementioned species have been implicated in bacillary necrosis, *V. tubiashii* has been identified as one of the most critical marine bacteria causing the well characterized adverse effect [8]. While *V. tubiashii* and *V. coralliilyitcus* exhibit similar virulence in the Eastern oyster, *V. coralliilyticus*-induced mortality is far greater than *V. tubiashii*-induced mortality in the Pacific oyster [9].

*V. coralliilyticus*, a Gram-negative, rod-shaped bacterium, is a well-known pathogen of coral, responsible for tissue lysis, bleaching, and drastic losses in coral reefs worldwide [10,11]. Moreover, this bacterium has been shown to infect fish [12] and bivalves, including the Pacific oyster [13,14]. Therefore, *V. coralliilyticus* could be considered as one of the major pathogens that causes economic damage by infecting various aquatic organisms.

In 2015, continuous mass mortalities in the Pacific oyster hatchery located in the southern area of Korea, led to huge commercial losses to hatchery farmers and aquaculture. For this reason, the present study was conducted to investigate the cause of the mass mortalities that continuously occur in Pacific oyster seedling production in Korea. We characterized the isolated *Vibrio* sp., analyzed its genome sequence to compare with previously reported strains, and investigated its virulence and antibiotic resistance genes.

## 2. Results and Discussion

### 2.1. Rule-Out and Identification of Causative Agent

The healthy larvae from Geoje island showed active mobility using their cilia, whereas the inactive larvae from Tongyeong were observed to be lethargic and had poor motility compared to healthy larvae. Almost all of the moribund and dead larvae showed specific clinical symptom of being infected by *V. coralliilyticus* and *V. tubiashii*, such as velum necrosis and adhesion of ciliates when observed under the microscope [15,16,17]. Except for feeding phytoplankton, no other parasitic protozoa were observed in the moribund larvae group or in the cultured water.

In general *V. coralliilyticus*, *V. tubiashii* and OsHV-1are known as main causative agents of the mass mortality of marine bivalve larvae [7,17,18]. Investigations made in this study on the mass mortality of Pacific oyster larvae specifically revealed that dead and moribund larvae experienced symptoms of bacillary necrosis and adhesion of ciliates. In the cases where larvae died from OsHV-1 infection, the dead larvae were not reported to have experienced the above-mentioned specific symptoms [19]. Molecular detection of OsHV-1 was diagnosed by conventional PCR using specific primer pairs. However, moribund and dead larvae showed a negative reaction, hence OsHV-1 could be dismissed as a major causative agent of the mass mortality. In contrast, Sugumar et. al. [7] and Tubiash et. al. [17] reported that dead larvae infected with *V. coralliilyticus* and *V. tubiashii* displayed these particular symptoms. Therefore, we focused on *V. coralliilyticus* and *V. tubiashii* infections that could be related to the mass mortality of Pacific oyster larvae in Korea. We conducted PCR using both *Vibrio* spp. specific primer pairs. PCR results using a primer pair specific to *V. tubiashii* were negative, indicating that these bacteria were not present in the dead and moribund larvae [15]. Whereas, a positive DNA band was detected in the DNA electrophoresis using *V. coralliilyticus* primer pair vcpAF/vcpAR, designed at zinc-metalloprotease gene region of *V. coralliilyticus* [20]. Therefore, it is very likely that the mass mortality can be associated with *V. coralliilyticus* infection.

Bacteria isolation from the moribund and dead larvae revealed a dominant gram-negative and curved rod-shaped strain. According to the PCR and sequencing of isolated bacterium results of the 16S rRNA region, over 99% of the sequence were similar to four corresponding genes of *V. coralliilyticus* (GenBank accession number: CP009617, CP009264, CP016556, and CP031472) from the BLAST search at NCBI. Therefore, the isolated strain in this study was considered to very likely be *V. coralliilyticus* and was designated as *V. coralliilyticus* SNUTY-1. To more clearly confirm the identification of the isolate, whole genome sequence analysis was performed.

### 2.2. Pathogenicity of Isolated V. Coralliilyticus SNUTY-1

**Figure 1:**
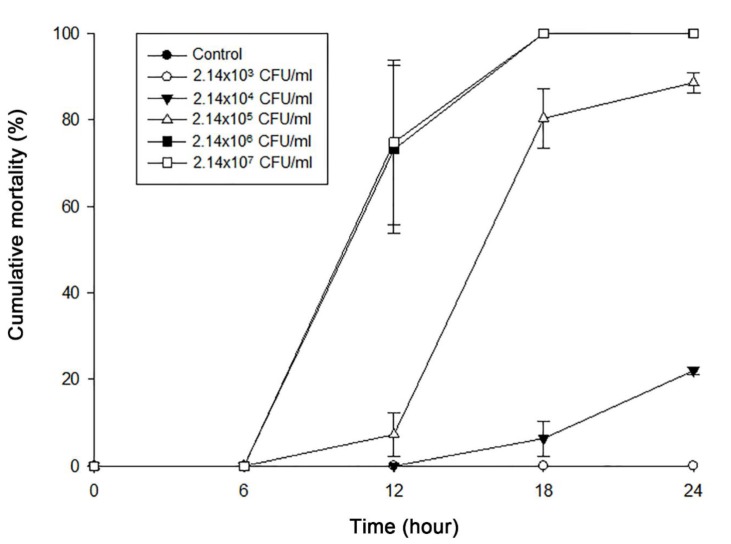
Pathogenicity of *V. coralliilyticus* SNUTY-1. Control indicates only larvae with FSS.

The healthy Pacific oyster larvae transported from Geoje island were shown to test negative for OsHV-1, *V. coralliilyticus*, and *V. tubiashii* using PCR. Therefore, we conducted a pathogenicity test with *V. coralliilyticus* SNUTY-1 using the Pacific oyster larvae (pathogen free) sampled from Geoje. All of the larvae in the negative control and the group treated with 2.14 × 10^3^ CFU/mL for 24 h survived. Larvae treated with 2.14 × 10^4^ CFU/mL and 2.14 × 10^5^ CFU/mL for 24 h resulted in 22.2 ± 0.84% and 88.65 ± 2.4% cumulative mortalities, respectively. At concentrations of 2.14 × 10^6^ CFU/mL and 2.14 × 10^7^ CFU/mL, 100% of the larvae died within 18 h (Figure 1). These results concurred with what has been previously reported for high pathogenic *V. coralliilyticus* strains [7,9,21]. In the pathogenicity test, moribund and dead larvae showed similar clinical signs with naturally infected samples. Moreover, an intense motile bacterial swarm was observed around the shell margin and cilia of the larvae. Inoculated *V. coralliilyticus* SNUTY-1 was re-isolated from all of the challenged larvae. The results of the experiment are confirmed as they coincide with Koch’s postulates.

### 2.3. Anti-Microbial Susceptibility and Biochemical Analysis of V. coralliilyticus SNUTY-1

The results of the antibiotic disk diffusion test are summarized in Table 1. *V. coralliilyticus* SNUTY-1 had strong antibiotic resistances to ampicillin, amoxicillin-clavulanate, ampicillin-sulbactam, piperacillin, cefepime, cefotaxime, cefoxitin, ceftazidime, and meropenem. *V. coralliilyticus* SNUTY-1 was confirmed to be resistant to 37.5% of the antibiotics tested in this study. In the biochemical test, 64 biochemicals were tested. The results from *V. coralliilyticus* SNUTY-1 were comparable with *V. coralliilyticus* 58, except for Ala-Phe-Pro-arylamidase and L-lactate alkalinization. Results of the biochemical test are presented in Supplementary Table S1. The VITEK^Ⓡ^2 System (bioMérieux^Ⓡ^, France) method is based on the ability of microbial substrates to identify and compare them with existing database [22]. In this study, we used VITEK^Ⓡ^2system to identify the isolated strain based on its biochemical characteristics. As pointed out earlier by O’Hara et al [23], it was impossible to identify the species using the data stored in the VITEK data base system. However, the result of the VITEK method (shown in Supplementary Table S1) could be considered as preliminary data for further biochemical characterization of *Vibrio* spp.

### 2.4. Genome of V. coralliilyticus SNUTY-1

General features of the *V. coralliilyticus* SNUTY-1 strain are summarized in Table 2. The fully assembled and closed *V. coralliilyticus* SNUTY-1 genome comprised of 5,842,676 bp, consisting of two chromosomes named Chr I (3,474,874 bp) and Chr II (1,976,676 bp), and two plasmids designated as pSNUTY1 (254,703 bp) and pSNUTY2 (136,423 bp). The two chromosomes exhibited similar G+C content (45.7% and 45.1%, respectively) and had similar percentages of the coding regions (87.9% and 87.6%, respectively). The annotated genome included 5527 genes, 5370 coding sequences, 37 rRNAs (5S, 16S, and 23S), 116 tRNAs, and four non-coding RNAs. The majority of the predicted tRNAs (*n* = 111), rRNA (*n* = 37), and ncRNA (*n* = 4) genes were encoded on Chr I, except for the 5 tRNA genes that were mostly on Chr II (Table 3). In addition, a total of four prophage regions (two intact and two incomplete) were identified (Supplementary Table S2).

The clusters of orthologous genes (COG) functional category analysis of *V. coralliilyticus* SNUTY-1 confirmed that Chr I had a higher percentage of genes associated with basic cellular functions compared to Chr II (Supplementary Figure S1a). Functional genes encoded on Chr I were primary involved in the COG categories of J (translation, ribosomal structure, and biogenesis), L (replication, recombination, and repair), D (cell cycle control, cell division, and chromosome partitioning), M (cell wall/membrane/envelope biogenesis), N (cell motility), U (intracellular trafficking, secretion, and vesicular transport), O (post-translational modification, protein turnover, and chaperones), C (energy production and conversion), F (nucleotide transport and metabolism), I (lipid transport and metabolism), and H (coenzyme transport and metabolism). On the other hand, Chr II possessed a higher percentage of genes involved in K (transcription), V (defense), T (signal transduction mechanisms), W (extracellular structures), X (mobilome, prophages, and transposons), G (carbohydrate transport and metabolism), E (amino acid transport and metabolism), P (inorganic ion transport and metabolism), and Q (secondary metabolites biosynthesis, transport, and catabolism). However, both of the two chromosomes contained genes involved in S (function unknown in COG database), and a match to 10.9% and 13.9% of the predicted genes on Chr I and Chr II, respectively, could not be found in the database. As expected, the search for a match in the database for most of the functional genes encoded on the plasmids pSNUTY1 (31.1%) and pSNUTY2 (47.4%) was unsuccessful (Supplementary Figure S1b).

**Figure 2:**
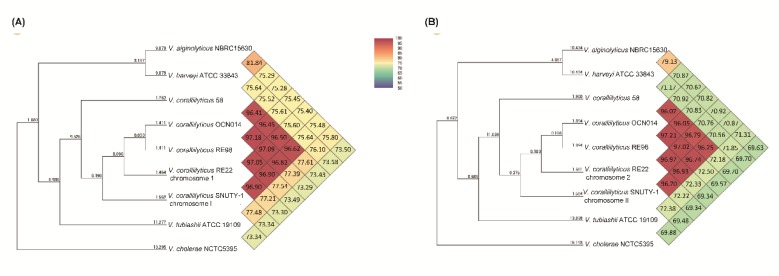
Phylogenetic position of *V. coralliilyticus* SNUTY-1 (A: ChrI, B: Chr II). Ortho ANI values calculated from the OAT software [29].

Currently, complete genomes of *V. coralliilyticus* OCN014 [24], RE98 [25], RE22 [26], and 58 [27], and *V. tubiashi* ATCC 19109 [28] are available in the GenBank database. Therefore, the OrthoANI algorithm [29] was applied to assess the overall genome similarities between *V. coralliilyticus* SNUTY-1 and the other related *Vibrio* strains. OrthoANI values were acquired and phylogenetic trees were constructed on the basis of the OrthoANI analysis of the four strains of *V. coralliilyticus* (58, OCN014, RE22 and RE98), the one strain of *V. tubiashii* (ATCC 19109), and other associated *Vibrio* spp. utilizing the orthologous average nucleotide identity tool. The result of phylogenetic trees on the basis of OrthoANI values for strain SNUTY-1 and other associated strains indicated that the Korean isolate, SNUTY-1, was closely related to the *V. coralliilyticus* strains (Figure 2). Moreover, the plasmids pSNUTY1 and pSNUTY2 showed no continuous sequence identity to other plasmid sequences in the GenBank database using BLAST search. Furthermore, plasmid pSNUTY1 was most similar to plasmids p337 (from *V. coralliilyticus* RE22, 99.2% identity) and plasmid p319 (from *V. coralliilyticus* RE98, 98.9% identity), with more than 50% coverage. However, plasmid pSNUTY2 was only similar to plasmid pLMB143 (from *V. campbellii* LMB29, 92.2% identity) with less than 10% coverage, and did not show any similarity to other plasmids found in *V. coralliilyticus* strains. These results strongly support the uniqueness of the Korean isolate from other sequenced *V. coralliilyticus* strains from different geographical origins, including strain 58 that was isolated from Japan [27]. 

Marine *Vibrio* species are known to produce toxic proteins such as cytolysins, exopolysaccharides, lipases, and proteases [30]. According to Hasegawa et al. [31], cytolysin/hemolysin (vthB/A) and extracellular metalloprotease (vtpA) have been described as virulence factors in *V. tubiashii*, and similar sequences were reported in *V. coralliilyticus* RE22 [24]. Similarly, the *V. tubiashii*-homologous cytolysin/hemolysin (vthB/A, 98.0% sequence identity) and metalloprotease genes (vtpA, 98.0% sequence identity) were encoded on Chr I and Chr II in *V. coralliilyticus* SNUTY-1, respectively. Moreover, another thirteen and six predicted cytolysin/hemolysin and metalloprotease, respectively, were found in the SNUTY-1 genome (Supplementary Table S3). Interestingly, we were able to detect the plasmid pSNUTY1-encoded hemolysinD gene, which was almost identical (>99%) to other plasmids (p337 and p319) found in *V. coralliilyticus* RE22 and RE98, thus suggesting that the virulent plasmid could also be associated with the pathogenicity of *V. coralliilyticus.* Additionally, the SNUTY-1 genome possessed antimicrobial-resistant genes involved in the resistance of β-lactams (MBL fold metallo-hydrolase and PBPs), fluoroquinolone (*qnrVv*), and phenicol (*catB3*). Moreover, the genome has been found to have homologues of tet(34) and tet(35), which has recently been described in tetracycline resistance of Vibrio species [32] (Supplementary Table S4). In contrast, results of the disc diffusion tests showed that the tested antibiotics were susceptible, except for β-lactams. The disc diffusion test was performed in this study use limited antibiotics from each antibiotic family following CLSI guideline. Although results of disc diffusion test did not match with genome analysis, tested antibiotics were chosen from various antibiotic family. Therefore, further study on resistant antibiotics based on antibiotic resistance genes should be carried out.

## 3. Materials and Methods

### 3.1. Sampling and Clinical Examination

**Figure 3:**
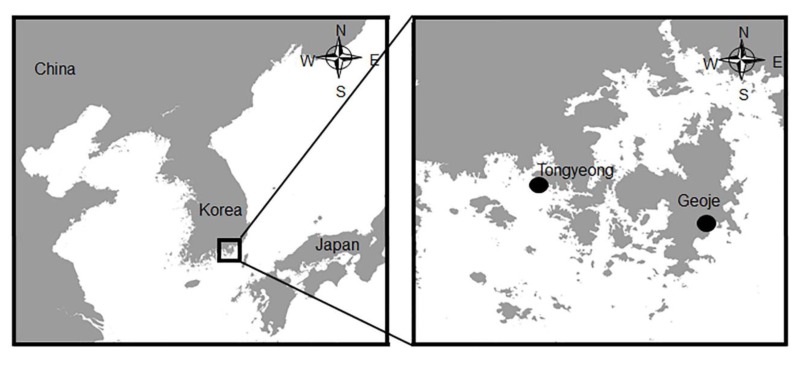
Sampling location of Pacific oyster larvae from Tongyeong (inactive larvae) and Geoje (healthy larvae) located at southern region of Korea.

Samples of five day-old dead and inactive Pacific oyster larvae (100–120 μm) that had sunk to the bottom of the culture tanks from a Pacific oyster hatchery at Tongyeong (34˚47’13”N, 128˚25’22”E), and healthy larvae (100–140 μm)from Geoje island (34˚47’07”N, 128˚32’40”E) were preserved at 4 °C and immediately transported to the Aquatic biomedicine laboratory, College of Veterinary Medicine, Seoul National University (Figure 3). Prior to the start of the experiment, larvae were washed three times using filtered and sterilized seawater (FSS; 33 psu, 0.22 μm, 121 °C for 15 min) in order to eliminate potential infections from other microorganisms. Washed samples were observed for clinical signs using an optical microscope (Olympus BX41, Olympus Optical Co., Ltd., Tokyo, Japan). In addition, moribund larvae and the seawater were moved to a plankton-counting chamber to verify the existence or nonexistence of parasitological organisms using an optical microscope.

### 3.2. Molecular Detection of Pathogen

Conventional polymerase chain reaction (PCR) was carried out to detect OsHV-1, *V. coralliilyticus*, and *V. tubiashii* that have been reported to be the main causative agents of mass mortalities in marine bivalve hatcheries worldwide. Fifty milligrams of moribund larvae were placed in a 1.5 mL centrifuge tube and the shells were broken by using a disposable tissue homogenizer. Total genomic DNA was extracted using a DNeasy Blood & Tissue Kit (Qiagen, CA, USA) following the manufacturer’s protocols. OsHV-1 was diagnosed using template DNA extracted from inactive larvae. PCR diagnosis of OsHV-1 DNA detection was conducted using C2/C6 primer pair, designed at the ORF-4 region following a previous study [18]. PCR for *V. coralliilyticus* and *V. tubiashii* were performed using specific primer pairs vcpAF/vcpAR, designed at the zinc-metalloprotease gene region of *V. coralliilyticus* [20] and protease and hemolysin gene region of *V. tubiashii* [15]. 

### 3.3. Bacteria Isolation and Identification

To isolate bacterium, fragmentized inactive larvae using a disposable tissue homogenizer plated on Marine Agar 2216 (BD Difco, New Jersey, USA) and Thiosulfate Citrate Bile Sucrose agar (BD Difco, New Jersey, USA) were incubated at 27 °C for 48 h. Colonies on both agar plates were re-spread onto Tryptic Soy Agar (TSA; BD Difco, New Jersey, USA) supplemented with NaCl (2.0% final concentration) and incubated at 27 °C for 24 h. The dominant single colony was then re-streaked onto TSA (2% NaCl) and incubated for an additional 24 h at 27 °C to obtain pure colonies. Gram staining was then performed using the isolated bacterium. Further, bacterial genomic DNA of the pure cultured bacteria was extracted using DNeasy Blood & Tissue Kit (Qiagen, CA, USA) following the manufacturer’s protocols. Analysis of 16S rRNA gene sequence was carried out using an ABI PRISM Big Dye TM Terminator Cycle Sequencing Kit (Applied BioSystem Inc., Massachusetts, USA) at Macrogen Inc. (Seoul, South Korea). Acquired 16S rRNA of bacterium sequence was subjected to BLAST search software supplied by the National Center for Biotechnology Information (NCBI). 

### 3.4. Pathogenicity Test of Isolated Bacterium

Healthy Pacific oyster larvae, 5 days old (100–140 μm) and preserved at 4 °C, were immediately transported from the oyster hatchery at Geoje to the laboratory. Healthy larvae were washed three times using FSS in order to eliminate other microorganisms. To confirm that the larvae were not infected by a pathogen, such as OsHV-1, *V. coralliilyticus* or *V. tubiashii*, PCR diagnosis was performed before the challenge test. Washed healthy larvae were placed into 6-well cell culture plates (SPL, Korea) with 10 mL of FSS. The density of the larvae was adjusted to 5 ± 2 larvae/mL. Cultured bacteria in Tryptic Soy Broth, adjusted to 2% NaCl (BD Difco, New Jersey, USA) at 27 °C for 24 h were centrifuged at 3000× *g* for 10 min and washed three times using FSS. Bacterial suspensions in FSS were adjusted from 2.14 × 10^3^ to 2.14 × 10^7^ colony-forming unit (CFU)/mL and inoculated into each wells for 24 h incubation at 27 °C. For the control, healthy larvae were placed into the wells without bacteria inoculation. We then investigated the cumulative mortality of the larvae for 24 h at 6 h intervals using an inverted microscope (Olympus CKX31, Olympus Optical Co., Ltd., Tokyo, Japan). The larvae without cilia movement and intestinal motility were verified to be dead by microscopic observation. The pathogenicity test was conducted in triplicate under the same conditions. For the challenge test, we attempted to re-isolate the inoculated bacterium from dead larvae following the same protocols as described above.

### 3.5. Anti-Microbial Susceptibility Test and Biochemical Analysis

The antimicrobial susceptibility test of the isolated bacterium was performed using 21 varieties of antibiotic disks (Oxoid, Hampshire, UK) that are recommended by the Clinical and Laboratory Standard Institute (CLSI) guideline. The list of antibiotics is summarized in Table 1. Standard disk diffusion method was conducted on Muller Hinton Agar (BD Difco, New Jersey, USA) at 27 °C for 24 h. Susceptibility and resistance were also determined according to the CLSI guideline [33]. *Escherichia coli* (ATCC 25922) was used in the experiment to clarify the strain criteria. For the biochemical analysis, VITEK^Ⓡ^2 System (bioMérieux^Ⓡ^, Marcy-l’Étoile, France) was performed using a gram-negative colorimetric identification card following manufacturer’s protocols. *V. coralliilyticus* 58, which is reported to be a strain with high virulence to Pacific oyster larvae [7,27], was also analyzed using the anti-microbial susceptibility test and biochemical test to compare with the isolated bacterium.

### 3.6. Genome Sequencing

The isolated bacterium was cultured overnight on TSA (2% NaCl) and then incubated at 27 °C for 24 h. Bacterial genomic DNA was extracted using a DNeasy Blood and Tissue Kit (Qiagen, CA, USA) following the manufacturer’s manual. Genome sequencing was conducted by Macrogen Inc. (Seoul, South Korea) by the PacBio RS II system (Pacific Biosciences, CA, USA), following construction of a 20 kb SMRTbell template library. The sequences generated (518,621,132 bp; 69,449 reads) were assembled using the Hierarchical Genome Assembly Process (HGAP) version 3.0 (https://github.com/PacificBiosciences/Bioinformatics-Training/wiki/HGAP), and the genome annotation was performed with the NCBI Prokaryotic Genome Annotation Pipeline (http://www.ncbi.nlm.nih.gov/books/NBK174280/).Genome annotation was conducted using the National Center of Biotechnology Information Prokaryotic Genome Annotation Pipeline (http://www.ncbi.nlm.nih.gov/books/NBK174280/), and PHASTER (http://phaster.ca/) analyzed to detect prophages. To assess the genomic relatedness to other *Vibrio* species, the average nucleotide identity was analyzed using OrthoANI (http://www.ezbiocloud.net/tools/orthoani). Potential virulence genes and antimicrobial resistance genes were preliminarily screened by searching against the Virulence Factor Database (http://www.mgc.ac.cn/VFs/) and the ARG-ANNOT database (http://en.mediterranee-infection.com/article.php?laref=283&titre=arg-annot-), respectively, and were then ultimately identified by manual comparisons with those reported for other *V. coralliilyticus* strains in the GenBank database.

## 4. Conclusions

In 2015, a continuous mass mortality event occurred at a Pacific oyster hatchery in Korea. We isolated *V. coralliilyticus*SNUTY-1 and confirmed that it was the causative agent through PCR analysis, whole genome sequence analysis, a challenge test, and clinical observation of signs such as velum necrosis and the adhesion of ciliates. A phylogenetic tree based on Ortho ANI values of SNUTY-1 showed that the Korean isolate was closely related to *V. coralliilyticus* strains. Results of a pathogenicity test revealed that SNUTY-1 was very pathogenic to Pacific oyster larvae. Whole genome sequence analysis of SNUTY-1 identified cytolysin/hemolysin, and extracellular metalloprotease encoded genes. Those genes might raise mortality in marine bivalve larvae. Thus, SNUTY-1 may potentially cause appreciable problems in marine bivalve hatcheries. In addition, genes that can encode for antibiotics resistance were also identified. The present study offers important insight into the biodiversity of the *Vibrio* sp. and provides valuable information for the study of virulence and antibiotic resistance factors, which will facilitate control of *V. coralliilyticus* in aquaculture. Further studies are required to determine appropriate treatments for preventing *V. coralliilyticus* infection–induced mass mortality events at marine bivalve hatcheries.

## 5. Culture Deposition and Nucleotide Sequence Accession Numbers

*V. coralliilyticus* SNUTY-1 was deposited at Korean Culture Center of Microorganisms (KCCM) under KCCM 43251. The complete genome sequence of *V. coralliilyticus* SNUTY-1 has been deposited in GenBank under accession numbers CP020453 (ChrI), CP020454 (ChrII), CP020455 (plasmid pSNUTY1), and CP020456 (plasmid pSNUTY2).

## Figures and Tables

**Table 1:** Result of antimicrobial susceptibility test of *V. coralliilyticus* strains (SNUTY-1 and 58).

Antibiotics	Strain		Antibiotics	Strain	
	SNUTY-1	58		SNUTY-1	58
Ampicillin	R ^*^	R	Meropenem	R	R
Amoxicillin-clavulanate	R	I ^*^	Amikacin	S ^*^	I
Ampicillin-sulbactam	R	R	Gentamicin	S	S
Piperacillin	R	R	Tetracycline	S	S
Piperacillin-tazobactam	S	I	Ciprofloxacin	S	S
Cefepime	R	R	Levofloxacin	S	S
Cefotaxime	R	R	Ofloxacin	S	S
Cefoxitin	I	R	Sulfonamides	S	S
Ceftazidime	R	R	Trimethoprim-sulfamethoxazole	S	S
Cefuroxime sodium	S	S	Chloramphenicol	S	S
Imipenem	S	S			

* R: Resistant; I: Intermediate; S: Susceptible.

**Table 2:** General features of *V. coralliilyticus* SNUTY-1 and MIGS mandatory information.

Items	Description
*Classification*	Domain *Bacteria*
	Phylum *Proteobacteria*
	Order *Vibrionales*
	Genus *Vibrio*
	Species *coralliilyticus*
	Strain: SNUTY-1
*General features*	
Gram stain	Gram negative
Cell shape	Curved rod
Motility	Motile with polar flagella
Temperature	4–37 °C
Pigmentation	Non-pigmented
*MIGS data*	
Investigation_type	Bacteria_archaea
Project_name	Genome sequence of *Vibrio coralliilyticus* SNUTY-1
Lat_lon	34˚47’13”N, 128˚25’22”E
Geo_loc_name	South Korea: Tongyeong
Collection_date	2015-04
Env_biome	landlocked sea [ENVO:00,000,219]
Env_feature	coastal water [ENVO:00,002,150]
Env_material	oyster [ENVO:02,000,079]
Num_replicons	4
Extrachrom_elements	2
Estimated_size	5,842,676
Ref_biomaterial	PMID: 9684317
Source_mat_id	KCCM 43251
biotic_relationship	Infectious (or commensal)
host	*Crassostrea gigas* (Pacific oyster) larvae
host_disease	bacillary necrosis
health_state	inactivated
Pathogenicity	Pathogenic in Pacific oyster (*Crassostrea gigas*)
Trophic_level	Chemoorganotroph
Rel_to_oxygen	Facultative anaerobic
Isol_growth_condt	PMID: 9684317
Seq_meth	PacBio RSII
Annot_source	GenBank
Finishing_strategy	Complete; 160× coverage, 4 contigs
*Genome assembly data*	
Assembly method	HGAP
Assembly name	HGAP algorithm ver. 3
Genome coverage	237×
Sequencing technology	PacBio RSII

**Table 3:** General features of *V. coralliilyticus* SNUTY-1 genome.

Attribute	Value			
	Chromosome I	Chromosome II	Plasmid pVs58	Plasmid pVs58
Size (bp)	3,474,874	1,976,676	254,703	136,423
Coding regions (%)	87.9	87.6	81.9	84.2
G+C content	45.7	45.1	49.3	44.7
Total genes	3358	1783	254	130
tRNA genes	111	5	-	-
rRNA genes	37	-	-	-
ncRNA genes	4	-	-	-

## Supplementary Materials

The following are available online at https://www.mdpi.com/2076-0817/9/3/206/s1, Figure S1a: Comparison of COG functional categories of the two chromosomes of *V. coralliilyticus* SNUTY-1, Figure S1b: COG functional categories of two plasmids of *V. coralliilyticus* SNUTY-1. Table S1: Results of biochemical test using VITEKⓇ2 System of *V. coralliilyticus* SNUTY-1 and 58, Table S2: The four prophage regions detected in the genome of *V. coralliilyticus* strain SNUTY-1, Table S3: Major virulence-related genes found in *V. coralliilyticus* strain SNUTY-1, Table S4: Major antimicrobial resistance related genes found in *V. coralliilyticus* strain SNUTY-1.

## References

[1] FAO FishStat (2019). Global Aquaculture Production for Species (Tonnes): Pacific Oyster. http://www.fao.org/fishery/topic/16140/en.

[2] Le Roux F., Zouine M., Chakroun N., Binesse J., Saulnier D., Bouchier C., Zidane N., Ma L., Rusniok C., Lajus A. (2009). Genome sequence of *Vibrio splendidus*: An abundant planktonic marine species with a large genotypic diversity. Environ. Microbiol..

[3] Di Salvo L.H., Blecka J., Zebal R. (1978). *Vibrio anguillarum* and larval mortality in a California coastal shellfish hatchery. Appl. Environ. Microbiol..

[4] Elston R.A. (1993). Infectious diseases of the Pacific oyster *Crassostrea gigas*. Annu. Rev. Fish Dis..

[5] Gómez-León J., Villamil L., Lemos M.L., Novoa B., Figueras A. (2005). Isolation of *Vibrio alginolyticus* and *Vibrio splendidus* from aquacultured carpet shell clam (*Ruditapes decussatus*) larvae associated with mass mortalities. Appl. Environ. Microbiol..

[6] Lodeiros C., Bolinches J., Dopazo C.P., Toranzo A.E. (1987). Bacillary necrosis in hatcheries of *Ostrea edulis* in Spain. Aquaculture.

[7] Sugumar G., Nakai T., Hirata Y., Matsubara D., Muroga K. (1998). *Vibrio splendidus* biovar II as the causative agent of bacillary necrosis of Japanese oyster *Crassostrea gigas* larvae. Dis. Aquat. Organ..

[8] Mersni-Achour R., Cheikh Y.B., Pichereau V., Doghri I., Etien C., Dégremont L., Saulnier D., Fruitier-Arnaudin I., Travers M.-A. (2015). Factors other than metalloprotease are required for full virulence of French *Vibrio tubiashii* isolates in oyster larvae. Microbiology.

[9] Richards G.P., Watson M.A., Needleman D.S., Church K.M., Häse C.C. (2015). Mortalities of Eastern and Pacific oyster larvae caused by the pathogens *Vibrio coralliilyticus* and *Vibrio tubiashii*. Appl. Environ. Microbiol..

[10] Ben-Haim Y., Thompson F.L., Thompson C.C., Cnockaert M.C., Hoste B., Swings J., Rosenberg E. (2003). *Vibrio coralliilyticus* sp. *nov.*, a temperature-dependent pathogen of the coral *c*. Int. J. Syst. Evol. Microbiol..

[11] Ben-Haim Y., Zicherman-Keren M., Rosenberg E. (2003). Temperature-Regulated Bleaching and Lysis of the Coral *Pocillopora damicornis* by the Novel Pathogen *Vibrio coralliilyticus*. Appl. Environ. Microbiol..

[12] Austin B., Austin D., Sutherland R., Thompson F., Swings J. (2005). Pathogenicity of *Vibrios* to rainbow trout (*Oncorhynchus mykiss*, Walbaum) and *Artemia* nauplii. Environ. Microbiol..

[13] Genard B., Miner P., Nicolas J.-L., Moraga D., Boudry P., Pernet F., Tremblay R. (2013). Integrative study of physiological changes associated with bacterial infection in Pacific oyster larvae. PLoS ONE.

[14] Kesarcodi-Watson A., Miner P., Nicolas J.-L., Robert R. (2012). Protective effect of four potential probiotics against pathogen-challenge of the larvae of three bivalves: Pacific oyster (*Crassostrea gigas*), flat oyster (*Ostrea edulis*) and scallop (*Pecten maximus*). Aquaculture.

[15] Elston R.A., Hasegawa H., Humphrey K.L., Polyak I.K., Häse C.C. (2008). Re-emergence of *Vibrio tubiashii* in bivalve shellfish aquaculture: Severity, environmental drivers, geographic extent and management. Dis. Aquat. Organ..

[16] Travers M., Achour R.M., Haffner P., Tourbiez D., Cassone A., Morga B., Doghri I., Garcia C., Renault T., Fruitier-Arnaudin I. (2014). First description of French *V. tubiashii* strains pathogenic to mollusk: I. Characterization of isolates and detection during mortality events. J. Invertebr. Pathol..

[17] Tubiash H.S., Chanley P.E., Leifson E. (1965). Bacillary necrosis, a disease of larval and juvenile bivalve mollusks I. Etiology and epizootiology. J. Bacteriol..

[18] Renault T., Arzul I. (2001). Herpes-like virus infection in hatchery-reared bivalve larvae in Europe: Specific viral DNA detection by PCR. J. Fish Dis..

[19] OIE (2018). Manual of Diagnostic Tests for Aquatic Animals: Chapter 2.4.5. Infection with Ostreid Herpesvirus 1 Microvariants. https://www.oie.int/index.php?xml:id=2439&L=0&htmfile=chapitre_ostreid_herpesvirus_1.htm.

[20] Pollock F.J., Morris P.J., Willis B.L., Bourne D.G. (2010). Detection and quantification of the coral pathogen *Vibrio coralliilyticus* by real-time PCR with TaqMan fluorescent probes. Appl. Environ. Microbiol..

[21] Kim H.J., Jun J.W., Giri S.S., Chi C., Yun S., Kim S.G., Kim S.W., Kang J.W., Han S.J., Kwon J. (2019). Application of the bacteriophage pVco-14 to prevent *Vibrio coralliilyticus* infection in Pacific oyster (*Crassostrea gigas*) larvae. J. Invertebr. Pathol..

[22] Stager C.E., Davis J.R. (1992). Automated systems for identification of microorganisms. Clin. Microviol. Rev..

[23] O’Hara C.M., Westbrook G.L., Miller J.M. (1997). Evaluation of Vitek GNI+ and Becton dickinson microbiology system crystal E/NF identification system for identification of members of the Family *Enterobacteriaceae* and other gram-negative glucose-fermenting and non-glucose-fermenting Bacilli. J. Clin. Microbiol..

[24] Ushijima B., Videau P., Poscablo D., Vine V., Salcedo M., Aeby G., Callahan S.M. (2014). Complete genome sequence of *Vibrio coralliilyticus* strain OCN014, isolated from a diseased coral at Palmyra atoll. Genome Announc..

[25] Richards G.P., Bono J.L., Watson M.A., Needleman D.S. (2014). Complete genome sequence for the shellfish pathogen *Vibrio coralliilyticus* RE98 isolated from a shellfish hatchery. Genome Announc..

[26] Spinard E., Kessner L., Gomez-Chiarri M., Rowley D.C., Nelson D.R. (2015). Draft genome sequence of the marine pathogen *Vibrio coralliilyticus* RE22. Genome Announc..

[27] Kim H.J., Kim J.H., Jun J.W., Giri S.S., Chi C., Yun S., Kim S.G., Kim S.W., Kang J.W., Jeong D.G. (2017). Complete genome sequence of *Vibrio coralliilyticus* 58, isolated from Pacific oyster (*Crassostrea gigas*) larvae. Genome Announc..

[28] Richards G.P., Needleman D.S., Watson M.A., Bono J.L. (2014). Complete genome sequence of the larval shellfish pathogen *Vibrio tubiashii* type strain ATCC 19109. Genome Announc..

[29] Lee I., Kim Y.O., Park S.-C., Chun J. (2016). OrthoANI: An improved algorithm and software for calculating average nucleotide identity. Int. J. Syst. Evol. Microbiol..

[30] Aguirre-Guzmán G., Mejia Ruíz H., Ascencio F. (2004). A review of extracellular virulence product of *Vibrio* species important in diseases of cultivated shrimp. Aquac. Res..

[31] Hasegawa H., Lind E.J., Boin M.A., Häse C.C. (2008). The extracellular metalloprotease of *Vibrio tubiashii* is a major virulence factor for Pacific oyster (*Crassostrea gigas*) larvae. Appl. Environ. Microbiol..

[32] Miranda C.D., Kehrenberg C., Ulep C., Schwarz S., Roberts M.C. (2003). Diversity of tetracycline resistance genes in bacteria from Chilean salmon farms. Antimicrob. Agents Chemother..

[33] Clinical and Laboratory Standards Institute (2015). Methods for Antimicrobial Dilution and Disk Susceptibility Testing of Infrequently Isolated or Fastidious Bacteria M45.

